# Association Between Arterial Stiffness, High Blood Pressure, and Hypertensive Phenotypes: Insights from the PAMELA Study

**DOI:** 10.3390/jcm14072230

**Published:** 2025-03-25

**Authors:** Fosca Quarti-Trevano, Cesare Cuspidi, Raffaella Dell’Oro, Pasquale Ambrosino, Guido Grassi

**Affiliations:** 1 Clinica Medica, School of Medicine and Surgery, University Milano-Bicocca, 20052 Monza, Italy; fosca.quarti@unimib.it (F.Q.-T.); raffaella.delloro@unimib.it (R.D.); 2 Department of Medicine and Surgery, University Milano-Bicocca, 20126 Milan, Italy; cesare.cuspidi@unimib.it; 3 Istituti Clinici Scientifici Maugeri IRCCS, Scientific Directorate of Telese Terme Institute, 82037 Telese Terme, Italy

**Keywords:** hypertension, arterial stiffness, endothelial function, inflammation, cardiovascular disability, chronic disease, exercise, rehabilitation, lifestyle risk reduction, outcome

## Abstract

Hypertension is a clinical condition associated with structural alterations in small, medium, and large arteries, also affecting target organs due to the mechanical effects of high blood pressure and shear stress. However, these vascular changes are also influenced by various inflammatory and neurohumoral mediators originating from the endothelium, the renin-angiotensin-aldosterone system, the neuroadrenergic system, and the perivascular fat. Specifically, chronic hypertension leads to vascular stretching, which triggers complex signaling pathways that promote vascular remodeling. The endothelium plays a crucial role in this process, as its function is impaired in hypertensive patients, leading to reduced nitric oxide-mediated vasodilation, increased vascular tone, and a proinflammatory and prothrombotic state. Along with structural changes, hypertension also triggers dynamic alterations in arterial distensibility and arterial wall properties, leading to increased arterial stiffness, which is strongly linked to cardiovascular outcomes and associated disability, as well as subsequent rehabilitation needs. Several non-invasive and highly reproducible methods are currently used to assess arterial stiffness, one of which is the cardio-ankle vascular index (CAVI). This article examines the association between arterial stiffness and high blood pressure, with a particular focus on the results of the Pressioni Arteriose Monitorate e Loro Associazioni (PAMELA) study. This study analyzes the determinants of arterial stiffness in the general population, the different hypertensive phenotypes affecting diurnal and nocturnal blood pressure profiles, and the impact of blood pressure control through antihypertensive treatment on arterial stiffness.

## 1. Introduction

Hypertension is commonly associated with structural changes in the large, medium, and small arteries and arterioles, as well as in target organs as a consequence of the mechanical effects of elevated blood pressure and shear stress values [1]. The structural cardiovascular alterations also depend, however, on the active influences of neurohumoral systems including the renin-angiotensin-aldosterone system, endothelins, neuroadrenergic influences, as well as substances generated in the perivascular fat and inflammatory mediators [1]. Chronically elevated blood pressure induces vascular stretching, which initiates complex signal transduction cascades leading to vascular remodeling [1]. The endothelium also exerts a fundamental regulatory action on the vascular tone. Its function is impaired in patients with hypertension, with a reduced nitric oxide-mediated vasodilation and increased vascular tone, coupled with a proinflammatory and prothrombotic state, as well as vascular remodeling [2].

Along with the above mentioned structural vascular modifications, hypertension also triggers dynamic alterations in arterial distensibility and/or changes in the arterial wall properties, i.e., modifications which favor the development and progression of an increased arterial stiffness. In recent years, the stiffness in large conduit arteries has been shown to bear a powerful independent association with cardiovascular outcomes and related disability [1]. This association represents the background for the growing interest for a direct, non-invasive, accurate, and reproducible method throughout which the assessment of arterial stiffness can be performed. The measurement of the cardio-ankle vascular index (CAVI) represents one of the simpler and affordable methods to assess arterial stiffness in clinical research [3].

In the present paper, we will review the main features of the association between arterial stiffness and high blood pressure, examining data collected in the Pressioni Arteriose Monitorate e Loro Associazioni (PAMELA) study, i.e., an epidemiological investigation carried out by our group in the outskirts of the Milan area, in northern Italy [4]. After briefly recalling the main features of the PAMELA study, this paper will focus on three pathophysiological and clinical aspects. First, we will analyze the main hemodynamic and non-hemodynamic determinants of arterial stiffness in the general PAMELA population. We will then examine the behavior of arterial stiffness in the different hypertensive phenotypes, which mainly affect the diurnal and nocturnal blood pressure profile. Emphasis will be finally given to the analysis of the impact of blood control and uncontrol by an antihypertensive drug treatment on arterial stiffness in the context of the PAMELA research project.

## 2. The PAMELA Study

The PAMELA study is an epidemiological investigation, which started more than 25 years ago, with the random selection of more than 2000 subjects from the general population living in Monza, a town located in the northeast surroundings of Milan, Italy [4]. The initial aim of this study was the definition of the normality of ambulatory and home blood pressure in the general population, which was information unknown at that time and crucial for employing out-of-office blood pressure values in daily clinical practice [4]. This was originally performed in the first survey, determining the upper limits of normality for home blood pressure and 24 h blood pressure, corresponding to clinical values of 140/90 mmHg defining the normotensive state [4]. Along with blood pressure values, other variables collected included metabolic and anthropometric variables, left ventricular mass, and atrial and aortic diameters [4]. The unique feature of this study was the reassessment of these variables throughout the years, with the first follow-up being performed between 2002 and 2003 and the second one between 2017 and 2018 [4]. This allowed for the collection of information on fatal and non-fatal cardiovascular events occurring during more than a quarter of a century, i.e., the longest follow-up ever performed. The PAMELA data allowed researchers to clarify the greater relevance that ambulatory blood pressure values have compared with clinical blood pressure in relation to target organ development (particularly at cardiac and renal level) and cardiovascular morbidity and mortality. The clinical relevance of the information derived from PAMELA data includes the impact different clinical hypertensive phenotypes have on cardiovascular fatal and non-fatal events. This applies in particular to white-coat hypertension, masked hypertension, nocturnal hypertension, and isolated systolic hypertension. Throughout the years, however, other important issues related to blood pressure normality as well as abnormality (high blood pressure) were addressed by the PAMELA data. They can be can be summarized under four main research areas [4]: (1) genetic profile and relationships with blood pressure values, (2) office, home, and ambulatory blood pressure data, including blood pressure variability, and relationships with cardiovascular risk factors and long-term prognosis for patients, (3) target organ damage with a particular focus on functional and structural alterations in the heart, and (4) the metabolic profile of the PAMELA cohort, with an analysis of the relationships between hemodynamic and metabolic variables. An additional area of investigation, which was carried out during the latest survey, refers to the cross-sectional evaluation of arterial stiffness in 519 subjects of the original population sample. For this aim, we assessed the subjects who were still alive after the first PAMELA survey between 2018 and 2019 via the measurement of the cardio-ankle vascular index (CAVI) using the VaSera System VS-2000 (Fukuda Denshi Co., Ltd., Tokyo, Japan) [3], and the overall stiffness properties of the main arterial vessels, including aorta, femoral, and tibial arteries. Relationships with different hypertensive phenotypes, daytime and nighttime ambulatory blood pressure profiles, as well as the main determinants of arterial stiffness were investigated. As far as the analysis of the data characterizing the different hypertensive phenotypes, it should be mentioned that the study power was > 98%, allowing us to detect differences in CAVI values between different patient groups with an alpha error amounting to 5%. A pathological increase in arterial stiffness was defined by a threshold value of CAVI > 9.0 m/s. This represents the value above the median obtained in the PAMELA population, in line with the abnormal cut-off proposed for the definition of high-risk patients by the Japanese Society for Vascular Failure [5].

## 3. Relationships Between CAVI and Other Variables

In the analysis of the data collected by assessing CAVI in the context of the third survey of the PAMELA study, several factors, classified as of hemodynamic or non-hemodynamic in nature, emerged as potential determinants of arterial stiffness. The sensitivity analysis of the data was performed to rule out any bias or inconsistency in the data collection.

### Arterial Stiffness and Demographic Variables

**Figure 1:**
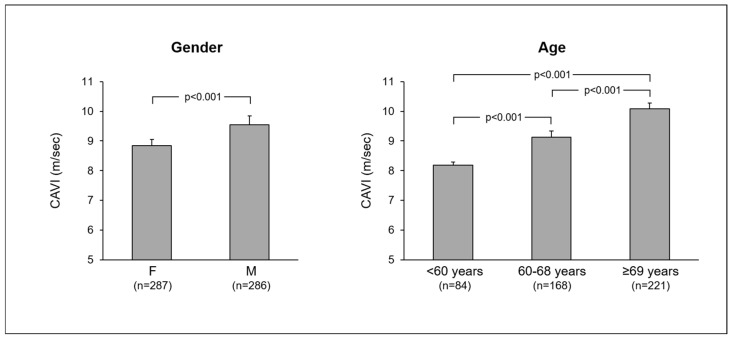
Mean ± standard errors in mean (SEM) values of cardio-ankle vascular index (CAVI) in the PAMELA population subdivided according to gender (**left panel**) and age (**right panel**). F: females, M: males. Numbers in parentheses refer to the subjects studied in each group. The figure was originally created using data from [9].

In the whole population, CAVI values showed a progressive significant age-related increase and displayed a significant gender-related difference, with male subjects being characterized by significantly higher CAVI values as compared to female individuals (Figure 1). Thus, confirming previous findings [6,7,8], data of the PAMELA study provide evidence on the age- and gender-related difference in arterial stiffness in a general population.

The main correlations between CAVI and hemodynamic and metabolic factors are shown in Table 1, which provides the level of statistical significance for data adjusted for confounders, such as age and gender.

As far as hemodynamic variables are concerned, significant relationships were detected between CAVI and systo-diastolic blood pressure values, when assessed by the doctor in the office, by the patients at home, or during a 24 h period via ambulatory monitoring. Taken together, these findings support the notion that arterial stiffness represents the main determinant of increasing absolute blood pressure (particularly the systolic component) in the essential hypertensive state [10]. Blood pressure variability, i.e., the blood pressure fluctuations occurring during the 24 h period related to the development of target organ damage and cardiovascular events [11], displays a different pattern as compared to absolute blood pressure values. Indeed, both the systolic and the diastolic components of the blood pressure variability phenomenon do not show any significant relationship with the CAVI. This is the case also when the analysis was performed while taking into account the so-called “residual variability”, an index reflecting the erratic systolic and diastolic blood pressure variations during a 24 h period [4].

Another hemodynamic variable, which has been examined in the PAMELA population for its possible relationships with the CAVI, is represented by the resting heart rate, which was assessed at the same time as the blood pressure measurement. At variance from some previous reports [1,12], which have shown significant associations between a high heart rate and elevated arterial stiffness at the level the carotid artery, the thoracic aorta, and the lower limbs, no relationship was detected in the PAMELA data between this hemodynamic variable and the CAVI. A possible explanation for the differences found between studies refers to the fact that the CAVI, in contrast to the pulse wave velocity, also includes value data related to the terminal aorta and the arteries in the arm, which, according to previous studies, are not associated with heart rate [1,4]. Considering that the heart rate may represent a marker, although indirect, of the sympathetic cardiovascular drive [2], the lack of any significant relationship with the CAVI suggests that neuroadrenergic influences may be less important than hemodynamic factors in modulating this variable.

Table 1 also shows that the CAVI is unrelated to the body mass index and waist-to-hip ratio. Furthermore, in contrast to previous reports [13,14], arterial stiffness does not display any significant relationship with metabolic variables, including total and HDL plasma cholesterol, plasma triglycerides, plasma glucose, and serum uric acid. It is likely that the degree of the metabolic alterations, which were of a mild degree in the PAMELA population, is responsible for the discrepancy of the findings between studies.

## 4. Arterial Stiffness in Hypertensive Phenotypes

**Figure 2:**
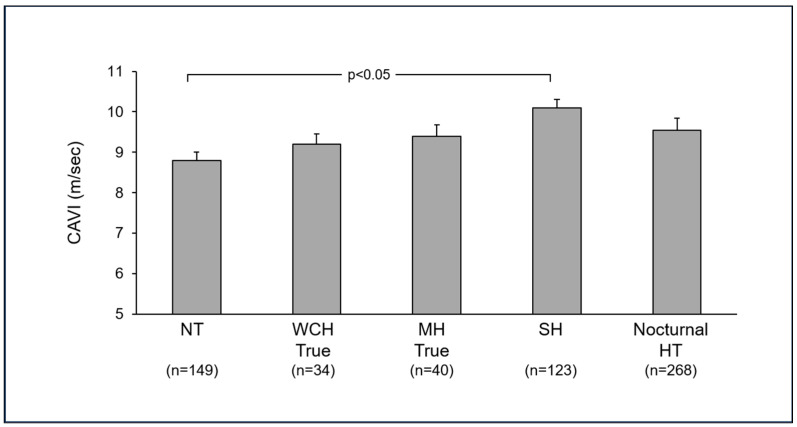
Mean ± standard error (SEM) values of cardio-ankle vascular index (CAVI) in the different normotensive and hypertensive phenotypes investigated in the PAMELA study. Numbers in parentheses refer to the subjects studied in each group. NT: normotensive subjects, age 65.4 ± 2.7 years, males 52.1%; WCH: white coat hypertensives, age: 64.3 ± 3.3 years, males 55.2%; MH: masked hypertensives, age: 62.3 ± 3.2 years, males 51.6%; SH: isolated systolic elderly hypertensives, age 68.4 ± 2.8 years, males 56.4%; Nocturnal HT: nocturnal hypertensives, age 66.1 ± 2.4 years, males 55.3%. The figure was originally created using data from [9].

In the PAMELA study, a number of hypertensive clinical phenotypes have been described. For many of them, data have been collected on arterial stiffness behavior by performing CAVI measurements. Their results, summarized in Figure 2, will be discussed in the following paragraphs.

### 4.1. White Coat Hypertension

This clinical phenotype, defined as the condition characterized by elevated office and normal out-of-office blood pressure values, has been quite extensively represented in the PAMELA study, with a prevalence amounting to 10–15% of the general population and 30–45% of the hypertensive one, depending on whether 24 h ambulatory blood pressure or home blood pressure measurements were taken into account for defining normal out-of-office blood pressure [4]. Recent analyses of the PAMELA data base have shown a frequent detection of this hypertensive phenotype in people with an increase in the body mass index, alterations in glucose and lipid metabolic profile, as well as the detection of the metabolic syndrome [4]. White-coat hypertension is not rarely associated with alterations in cardiovascular structure and function, with the identification of left ventricular hypertrophy, left ventricular diastolic dysfunction, and carotid intima-media thickening, with an adverse impact on cardiovascular risk profile [15]. The assessment of the CAVI has also shown an increase in arterial stiffness (Figure 2), which is associated with echocardiographic evidence of left ventricular remodeling and left ventricular hypertrophy [9]. A further subanalysis of the data collected in the PAMELA study allowed us to identify the so-called “partial white-coat” phenotype, characterized by an increase in office blood pressure with a normality of only one of the two out-of-office blood pressure measurements (home or 24 h ambulatory). Despite this selective alteration, the assessment of the CAVI has also documented a significant increase in arterial stiffness in this peculiar clinical phenotype [9].

### 4.2. Masked Hypertension

Similarly to what has already been described for white-coat hypertension, the opposite condition, i.e., masked hypertension, is also characterized by normal office but elevated home and 24 h blood pressure values; this characteristic is also quite frequently detected, affecting about 10% to 12% of the PAMELA population study [4]. This phenotype is also characterized by an increased cardiovascular risk, which is linked to the detection of target organ damage, as assessed by cardiac and carotid ultrasonographic examination [9]. By assessing arterial stiffness using the CAVI, increased values have also been detected (Figure 2), with evidence of abnormal values in the so-called “partial masked” phenotype as well, which is characterized by office blood pressure values in the normotensive range, coupled with abnormal values of only one of the out-of-office blood pressure measurements (either home or 24 h blood pressure) [9]. The recent detection of combined alterations in arterial stiffness and left ventricular remodeling [9] emphasizes the need for a parallel search of vascular and cardiac organ damage in this phenotype as well.

### 4.3. Isolated Systolic Hypertension

The clinical relevance of this phenotype is growing, given the evidence that in people aged 60 years and older, this is by far the most common form of clinical hypertension [16]. Its elevated prevalence is associated with a higher incidence of target organ damage, cardiovascular outcomes, and related disability [16]. Findings from the PAMELA study have documented the frequent report in this clinical phenotype of a pathological increase in arterial stiffness, with values significantly greater than those found not only in the pure normotensive state but also in the other hypertensive phenotypes (Figure 2) [9].

### 4.4. Nocturnal Hypertension

In the PAMELA population, about one-third of hypertensive participants were found to fulfill the diagnostic criteria for this clinical phenotype [4]. Patients with nocturnal hypertension (a hypertensive phenotype displaying a blood pressure elevation restricted to the nighttime period) are characterized by older age, greater prevalence of diabetes, higher circulating plasma levels of creatinine, uric acid, and homocysteine than nocturnal normotensive subjects. Furthermore, a relationship between isolated nocturnal hypertension and target organ damage has been documented in recent years [17]. These findings are in line with the notion that nighttime blood pressure is superior to daytime blood pressure in predicting subclinical cardiac and extra-cardiac organ damage and, more importantly, the risk of fatal and non-fatal cardiovascular events [18]. This is the case also for arterial stiffness evaluation, with evidence of CAVI values greater than those found in normotensive controls. An additional analysis of the PAMELA data related to the nighttime blood pressure profile provides evidence that subjects displaying a lack of blood pressure reduction at night (so called “non-dippers”) or those paradoxically showing a blood pressure elevation during the nighttime period (so-called “reverse dippers”) reveals CAVI values significantly higher than those reported in subjects displaying the physiological nocturnal blood pressure reduction (so-called “dippers”).

## 5. Arterial Stiffness and Blood Pressure Control

**Figure 3:**
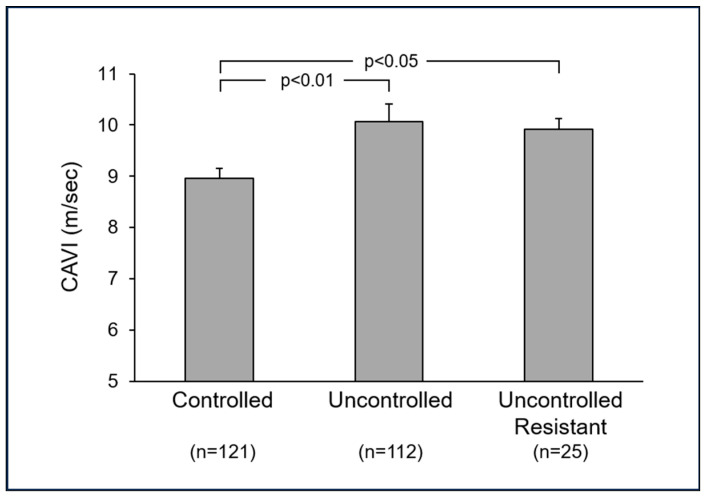
Mean ± standard errors in mean (SEM) values of cardio-ankle vascular index (CAVI)) in hypertensive patients displaying blood pressure values controlled or uncontrolled by antihypertensive drug treatment and in resistant hypertensives. Numbers in parentheses refer to the subjects studied in each group. The figure was originally created using data from [15].

Data collected in the context of the PAMELA research project have shown that in the treated fraction of the hypertensive population included in this study, systolic and diastolic office blood pressure control using an antihypertensive drug treatment amounted, on average, to 29.9% and 41.5%, respectively, with the corresponding values for 24 h ambulatory blood pressure being 50.8% and 64.0% [4]. When the evaluation of the CAVI was performed in these subgroups of treated hypertensive patients, significant differences in arterial stiffness were found, confirming the findings obtained in previous studies [18,19]. Indeed, the patients with an uncontrolled hypertensive phenotype display CAVI values significantly greater than those detected in individuals displaying clinic and ambulatory blood pressure values which are well-controlled by antihypertensive drugs (usually as combination treatment) (Figure 3). The CAVI differences cannot be ascribed to dissimilarities between patient groups concerning the use of antihypertensive drugs with vasodilating properties, such as calcium channel blockers, which are known to exert direct effects on arterial stiffness properties [20].

CAVI measurements were also performed in the so-called drug-resistant hypertensive patients, i.e., subjects in which the treatment of elevated blood pressure values by three or more antihypertensive agents (including a diuretic drug), employed at the full daily dosage, failed to achieve blood pressure control [20]. The results shown in Figure 3 document that, similarly to what was found in hypertensives with elevated blood pressure values uncontrolled by pharmacological treatment, in these patients, the CAVI displays values significantly greater than those detected in the pure normotensive control group [15]. Taken together, these findings support the concept that alterations in arterial stiffness are commonly detected in both uncontrolled and resistant hypertensive patients. As discussed in the next paragraph, in this latter group of patients, the CAVI assessment may provide insights on the clinical efficacy of therapeutic interventions, such as renal denervation [20].

Finally, some limitations of the studies based on the CAVI assessment in hypertension should be recognized. One limitation is that this arterial index is closely dependent on blood pressure values, which by definition are different in controlled, uncontrolled, and resistant hypertensive states. Thus, whether the CAVI differences described in the various hypertensive phenotypes are dependent only on hemodynamic factors or also on other non-hemodynamic variables, such as muscle cell reactivity and contractile process, remains to be defined.

## 6. Conclusions

The data reviewed in this paper strongly support the notion that a common pathophysiological hallmark of essential hypertension is an alteration in arterial stiffness, which can be detected in different clinical phenotypes. This finding has clinical relevance, considering that arterial stiffness is a marker of reduced distensibility and contractility of the arterial wall, retaining independent prognostic relevance in different cardiovascular diseases [1]. Antihypertensive pharmacological treatment can at least in part reverse these alterations, but only in the clinical conditions in which office and out-of-office measurements display a full and stable blood pressure control by an antihypertensive drug treatment. Evidence has been provided that drugs acting on the renin-angiotensin system as well as calcium channel blockers exert beneficial effects by reducing arterial stiffness, while classic beta blocking agents without vasodilating properties and thiazide diuretics may have opposite effects [20]. Finally, a unique feature of the CAVI assessment is represented by the finding that patients with arterial stiffness < 9 m/s display better blood pressure-lowering effects induced by bilateral renal nerve ablation [20]. This suggests that the CAVI assessment may also be relevant for predicting the clinical outcome of a procedure which is becoming more and more common in hypertension treatment.

## Figures and Tables

**Table 1:** Relationships between cardio-ankle vascular index (CAVI) and other variables.

Variable	r Value	Adjusted *p*-Value
Body mass index (kg/m^2^)	−0.1799	0.3323
Waist circumference (cm)	−0.05034	0.2184
Office SBP (mmHg)	0.28363	0.0003
Office DBP (mmHg)	0.11589	0.0024
Office HR (b/min)	0.03674	0.6693
Home SBP (mmHg)	0.28937	0.0003
Home DBP (mmHg)	0.0732	0.0035
Home HR (b/min)	0.00982	0.5401
24 h SBP (mmHg)	0.23004	0.0005
24 h DBP (mmHg)	0.12726	0.0002
24 h SBP SD	0.02594	0.5588
24 h DBP SD	0.02913	05116
24 h HR (b/min)	−0.02834	0.5676
Total cholesterol (mg/dL)	−0.09252	0.6976
HDL cholesterol (mg/dL)	−0.02894	0.6684
Serum glucose (mg/dL)	0.12326	0.1259
Type 2 diabetes (%)	0.13813	0.1049
Triglycerides (mg/dL)	0.03829	0.5157
Uric acid (mg/dL)	0.02524	0.5536
Serum creatinine (mg/dL)	0.03636	0.3835

Correlation coefficients (r) and adjusted *p*-values are corrected for age and gender. SBP: systolic blood pressure; DBP: diastolic blood pressure; HR: heart rate; SD: standard deviation. The table was originally created using data from [9].

## Data Availability

No datasets were generated or analyzed during the current study.

## References

[1] Chirinos J.A., Segers P., Hughes T., Townsend R. (2019). Large-artery stiffness in health and disease: JACC State-of-the-Art Review. J. Am. Coll. Cardiol..

[2] Fortini F., Vieceli Dalla Sega F., Marracino L., Severi P., Rapezzi C., Rizzo P., Ferrari R. (2021). Well-known and novel players in endothelial dysfunction: Updates on a notch(ed) landscape. Biomedicines.

[3] Saiki A., Ohira M., Yamaguchi T., Nagayama D., Shimizu N., Shirai K., Tatsuno I. (2020). New horizons of arterial stiffness developed using cardio-ankle vascular index (CAVI). J. Atheroscler. Thromb..

[4] Grassi G., Quarti-Trevano F., Dell’Oro R., Cuspidi C., Mancia G. (2021). The PAMELA research project: A 25-year long journey. Panminerva Med..

[5] Tanaka A., Tomiyama H., Maruhashi T., Matsuzawa Y., Miyoshi T., Kabutoya T., Kario K., Sugiyama S., Munakata M., Ito H. (2018). Physiological diagnosis criteria for vascular failure committee. Physiological diagnostic criteria for vascular failure. Hypertension.

[6] Lu Y., Pechlaner R., Cai J., Yuan H., Huang Z., Yang G., Wang J., Chen Z., Kiechl S., Xu Q. (2020). Trajectories of age-related arterial stiffness in chinese men and women. J. Am. Coll. Cardiol..

[7] Lu Y., Kiechl S.J., Wang J., Xu Q., Kiechl S., Pechlaner R., Global Pulse Wave Velocity Study Group (2023). Global distribution of age- and sex-related arterial stiffness: Systematic review and meta-analysis of 167 studies with 509,743 participants. EBioMedicine.

[8] Fantini F., Giani A., Trentin M., Rossi A.P., Zoico E., Mazzali G., Micciolo R., Zamboni M. (2022). The correlation of arterial stiffness parameters with aging and comorbidity burden. J. Clin. Med..

[9] Cuspidi C., Facchetti R., Gherbesi E., Quarti-Trevano F., Vanoli J., Mancia G., Grassi G. (2024). Ambulatory blood pressure phenotypes, arterial stiffness, and cardiac remodeling. Am. J. Hypertens..

[10] Boutouyrie P., Chowienczyk P., Humphrey J.D., Mitchell G.F. (2021). Arterial stiffness and cardiovascular risk in hypertension. Circ. Res..

[11] Steinsaltz D., Patten H., Bester D., Rehkopf D. (2025). Short-term and mid-term blood pressure variability and long-term mortality. J. Am. Coll. Cardiol..

[12] Kase M., Iijima T., Niitani T., Sagara M., Sakurai S., Tomaru T., Jojima T., Usui I., Aso Y. (2022). Relationship between reduced heart rate variability and increased arterial stiffness evaluated by cardio-ankle vascular index in people with type diabetes. Diabetol. Int..

[13] Wang H., Liu J., Zhao H., Zhao X., Li L., Shi H., Zhan S., Liu J. (2015). Relationships between cardio-ankle vascular index and plasma lipids in hypertension subjects. J. Hum. Hypertens..

[14] Nagayama D., Watanabe Y., Saiki A., Shirai K., Tatsuno I. (2018). Lipid parameters are independently associated with cardio-ankle vascular index in healthy japanese subjects. J. Atheroscler. Throm.

[15] Townsend R.R., Cohen J.B. (2024). White-coat hypertension & cardiovascular outcomes. Curr. Hypertens. Rep..

[16] AlGhatrif M., Lakatta E.G. (2015). The conundrum of arterial stiffness, elevated blood pressure, and aging. Curr. Hypertens. Rep..

[17] Kim H.S., Shin C., Kim S., Kim J.S., Lim A.Y., Seo H.S., Lim H.E., Sung K.C., Cho G.Y., Lee S. (2022). Prevalence of isolated nocturnal hypertension and development of arterial stiffness, left ventricular hypertrophy, and silent cerebrovascular lesions: The KoGES (Korean Genome and Epidemiology Study). J. Am. Heart Assoc..

[18] Kario K., Hoshide S., Mizuno H., Kabutoya T., Nishizawa M., Yoshida T., Abe H., Katsuya T., Fujita Y., Okazaki O. (2020). Nighttime blood pressure phenotype and cardiovascular prognosis: Practitioner-based nationwide JAMP study. Circulation.

[19] Kawabata T., Kubozono T., Ojima S., Kawasoe S., Akasaki Y., Salim A.A., Ikeda Y., Miyata M., Takenaka T., Ohishi M. (2022). Insufficient blood pressure control is independently associated with increased arterial stiffness. Hypertens. Res..

[20] Mancia G., Kreutz R., Brunström M., Burnier M., Grassi G., Januszewicz A., Muiesan M.L., Tsioufis K., Agabiti-Rosei E.A., Algharably E.A.E. (2023). 2023 ESH guidelines for the management of arterial hypertension. J. Hypertens..

